# Music, health, and well-being

**DOI:** 10.3402/qhw.v8i0.21780

**Published:** 2013-08-07

**Authors:** Marie Strand Skånland

Music and health as a field of research has flourished in the recent years, although the belief in a link between music and health is not new. In fact, the idea about music as salutary dates all the way back to the ancient Greeks and Pythagoras (Ruud, this volume; West, 2000). Ansdell (2013) comments:

But of course this link between people, music, and health/wellbeing is hardly new. It is a perennial knowledge that has been understood and practised in very varied ways across history—and varies still across contemporary social and cultural sites and traditions (Horden, 2000; Gouk, 2000; Gioia, 2006). The appreciation that music helps has been—and still is—either too obvious to mention, or else something forgotten and strange; is either central to normal cultural practice, or else marginal; is either mostly a professional expertise, or else an everyday lay practice. (p. 4)

Music, health, and well-being is a broad field that encompasses different areas, such as music therapy, music medicine, and music as self-care in everyday life (see Bonde, 2011; MacDonald, this volume; Ruud, 2008). Although some people experience music as nearly “magical” (Skånland, 2012), music by itself normally does very little (Ansdell, 2013; DeNora, 2000). Instead, we might understand its impact to derive from the *relationship* between music and the person. In other words, music is not merely a stimulus that “acts” on listeners; it offers certain opportunities to the people who engage with it and provides its benefits based on the ways in which these people interact with or *appropriate* it (Clarke, 2003; DeNora, 2000; Skånland, 2012). If we seek to understand these processes, then, we must recognize individuals’ competences related to the use of music in their self-care (see Ruud, this volume). But we must also account for the research-based expertise that music therapists can offer to people with special needs (see Gilbertson, this volume; McFerran & Shoemark, this volume; Stensæth, this volume). Ansdell (2013) suggests that we view the field of music and health as a continuum between the applications and roles of music in everyday life and those of music in “specialist life” (i.e., music therapy). The variety of emphases in the present issue, from the use of music as self-care in everyday life to the use of music in a range of therapeutic settings, will hopefully offer the reader some insight into this continuum.

In addition, thinking in terms of a continuum can also be valuable when it comes to health, as suggested by Antonovsky (1979, 1987). Rather than framing health and disease as a dichotomy, Antonovsky positions all of us on a continuum between these two theoretical poles. In looking at what facilitates an individual's movement towards the salutary end of this continuum (instead of merely investigating the factors that contribute to a specific disease), we might generate new ways of decreasing human suffering on an expansive scale (Antonovsky, 1979). This *resource-oriented* view of health is also fruitful with regard to the relationship between music and health. Music, whether introduced into a therapeutic relationship or an individual's private life, can strengthen people's already existing resources and add to their sense of well-being, quality of life, and general positive mental health (Bonde, Ruud, Skånland, & Trondalen, 2013; DeNora, 2007; Ruud, 2008).

In this special issue on music, health, and well-being, MacDonald maps the current field of research, introducing five areas that encompass a perspective on music and health while also introducing a number of qualitative research projects focused on exploring music interventions. Ruud looks at the therapeutic possibilities of everyday uses of music through six narratives about musical self-care and identifies six generative factors that may contribute to the health benefits of music. Skånland looks further into music's impact on the everyday lives of regular users of MP3 players, exploring the role of this mobile music technology in people's affect regulation using qualitative interviews. McFerran and Shoemark, Stensæth, and Gilbertson, respectively, provide excellent examples of different uses of music in therapeutic settings: McFerran and Shoemark discuss music therapy in a pedagogical context, examining the case of a young man with profound disabilities and his relationship with his music therapist; Stensæth describes the development and use of new technology for severely disabled children and their families, exploring the implications of musical and interactive tangibles used as health-promoting implements; and Gilbertson explores new methodologies for investigating relationships in music therapy improvization, using multi-data qualitative research methods to look at the meaning allocated to an episode of music improvization in early neurosurgical rehabilitation by a teenage boy with severe traumatic brain injury and his music therapist. DeNora, finally, enters the debate concerning quantitative assessment through a response to calls for evidence-based medicine and evidence-based practice, arguing instead for an ecological approach to music and health research. In all, these articles offer different perspectives on the already broad but still expanding research field of music, health, and well-being and will hopefully leave the reader with new insights into music's possibilities.

*Marie Strand Skånland* PhD
